# A Study of Trimethylsilane (3MS) and Tetramethylsilane (4MS) Based α-SiCN:H/α-SiCO:H Diffusion Barrier Films

**DOI:** 10.3390/ma5030377

**Published:** 2012-03-02

**Authors:** Sheng-Wen Chen, Yu-Sheng Wang, Shao-Yu Hu, Wen-Hsi Lee, Chieh-Cheng Chi, Ying-Lang Wang

**Affiliations:** 1 Department of Materials Engineering, National Cheng Kung University, Tainan 70101, Taiwan; E-Mail: swcheni@tsmc.com; 2 Department of Electrical Engineering, National Cheng Kung University, Tainan 70101, Taiwan; E-Mails: yswange@tsmc.com(Y.-S.W.); n2895153@mail.ncku.edu.tw (S.-Y.H.); l9466601@gmail.com (C.-C.C.); 3 Institute of Lighting and Energy Photonics, National Chiao Tung University, Hsinchu 30050, Taiwan; E-Mail: ylwang@tsmc.com

**Keywords:** SiC(N), interface state, bonding configuration

## Abstract

Amorphous nitrogen-doped silicon carbide (α-SiCN:H) films have been used as a Cu penetration diffusion barrier and interconnect etch stop layer in the below 90-nanometer ultra-large scale integration (ULSI) manufacturing technology. In this study, the etching stop layers were deposited by using trimethylsilane (3MS) or tetramethylsilane (4MS) with ammonia by plasma-enhanced chemical vapor deposition (PECVD) followed by a procedure for tetra-ethoxyl silane (TEOS) oxide. The depth profile of Cu distribution examined by second ion mass spectroscopy (SIMs) showed that 3MS α-SiCN:H exhibited a better barrier performance than the 4MS film, which was revealed by the Cu signal. The FTIR spectra also showed the intensity of Si-CH_3_ stretch mode in the α-SiCN:H film deposited by 3MS was higher than that deposited by 4MS. A novel multi structure of oxygen-doped silicon carbide (SiC:O) substituted TEOS oxide capped on 4MS α-SiC:N film was also examined. In addition to this, the new multi etch stop layers can be deposited together with the same tool which can thus eliminate the effect of the vacuum break and accompanying environmental contamination.

## 1. Introduction

As Ultra Large-Scale Integrated circuits (ULSIs) have been reduced to ultra deep submicron dimensions, signal propagation delay, cross talk, and power consumption have drastically increased due to the parasitic capacitance of intra-level interconnection. Copper (Cu) interconnection and low dielectric *k* (*k* < 3.0) materials have been used from 0.13 um technology to reduce the RC delay time, interconnection resistance and interlayer capacitance [1,2]. However, the Cu interconnection has several disadvantages for application in process technology. For example, a Cu film is easily oxidized, and Cu atoms or ions easily diffuse into low *k* interlayer dielectrics by thermal annealing or with electric fields [3]. Thus, it is desirable to develop new materials with a lower *k*-value to further reduce the effective dielectric constant of the Cu interconnect system [4,5]. It is also known that Cu is a serious source of contamination for both silicon and silicon dioxide. To prevent Cu from diffusion into the dielectric material, Cu must be sealed using diffusion barriers. A dielectric diffusion barrier layer must be deposited on Cu wires to seal the Cu and to serve as the etch stop layer during the via etch of the next metal layer [6,7]. In 90 nm and 65 nm technology, dual layers of composite α-SICN:H/TEOS oxide were used as a Cu diffusion barrier. The role of the first layer, α-SICN:H, is as a Cu diffusion barrier and etch stop layer. The second layer TEOS oxide is used to prevent PR poisoning. In this work, we investigate the dielectric constants between measurement and theoretically calculated, the chemical bonding configuration, and the potential of the Cu diffusion barrier with a bi-layer structured α-SiCN:H/TEOS barrier [8]. 

## 2. Experimental Section

The SiCO- or SiCN-base films on a copper layer have been widely used in the copper dual damascene process as a copper ion barrier layer [9,10]. Moreover the influences of the etching-stop-layer, silicon carbide (SiC) barrier cap layer, and deposition process on electro migration (EM) and stress migration (SM) have been reported [4,11]. The copper and barrier layer interface is the dominant path for copper migration [5,11,12]. One of the reliability issues in Cu metallization is dielectric degradation caused by Cu ion penetration. Copper could rapidly drift into silica-based low-k dielectrics during bias-temperature stressing [13]. In this article, it was demonstrated that a novel multi structure of an oxygen-doped SiC and nitrogen-doped SiC deposited at 350 °C showed excellent Cu blocking capability.

The 3MS and 4MS SiC samples were prepared at 350 °C by 300 mm Producer SE^TM^ PECVD of Applied Material and 300 mm Vector TM PECVD of Novellus, respectively. Sample preparations followed experimental conditions on 300 mm p-type silicon wafers. The deposition conditions are listed in Table 1.

**Figure 1:**
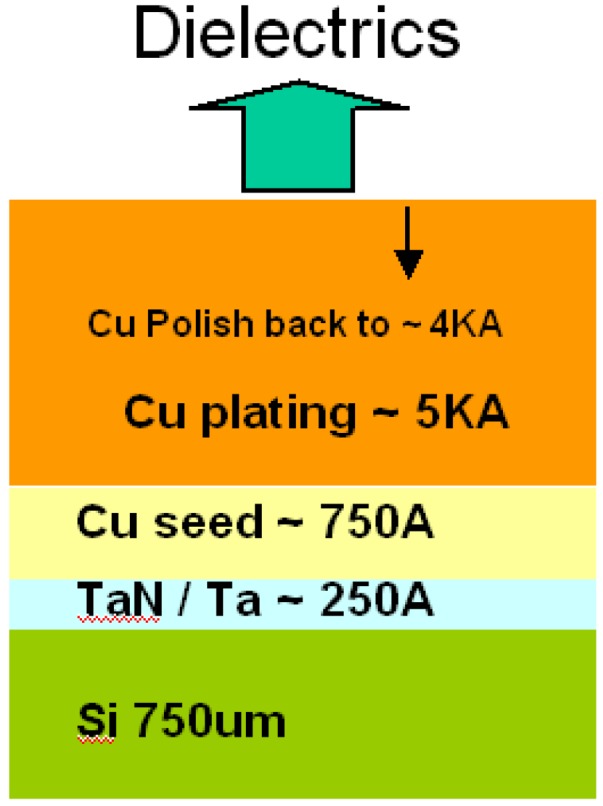
Sample prepared film stacked.

(1) Optical thickness and k test method:
  300 mm KLA-Tencor FX-100, 633 nm, and Quantox measured thickness and k value, respectively.
(2) FTIR test method:
  FTIR test method was carried out by 300 mm BIO-RAD SS-3300 spectrometer of Accent. Sample preparations followed experimental conditions on 300mm p-type silicon wafer.
(3) SIMs:
  Second Ion Mass Spectroscopy was used to measure the depth profile of Cu. Samples were prepared, as figure 1, by physical vapor deposition 250 A TaN/Ta on a 750 µm bare silicon wafer. In order to provide copper electrochemical plating 5000A, the wafers were deposited with a 750A copper seed layer by physical vapor deposition. After chemical mechanical polishing back to 4000A, all dielectric samples were deposited.

**Table 1:** Deposition condition of trimethylsilane (3MS) or tetramethylsilane (4MS)-base films.

Sample identification	4MS		3MS		TEOS ^a^
	N a-SiCN:H	N a-SiCO:H	A a-SiCN:H	SiCO:H	PEOX
Pressure (torr)	2.0~3.0		3.0~4.0		1.5
HFRF ^b^	550~600		450~600		200
LFRF ^b^	900	-	0	80	800
N/Si ratio	2.06	-	1.84	-	-
C/Si ratio	-	6.55	-	-	-
O/Si ratio	-	-	-	2.0	12,000

^a^: TEOS: tetraethoxyl silane; ^b^: HFRF: High Frequency Radio Frequency; LFRF: Low Frequency Radio Frequency.

## 3. Results and Discussion

Table 2 shows the measurements of optical thickness, electrical thickness and dielectric constant *k* of the samples, deposited on bare Si wafers. It can be seen that all the dielectric constants *k* of the samples were comparable with previous literature. However, comparing the A α-SiCN:H with N α-SiCN:H films, the dielectric *k* of N α-SiCN:H film is higher than A α-SiCN:H film by about 15%. It is worth noting that the main precursors of A α-SiCN:H and N α-SiCN:H films are 3MS and 4MS, respectively, and the structure of N α-SiCN:H film in principle contains more methyl bonds (-CH_3_) than A α-SiCN:H film which should lead to a lower dielectric constant. However, as illustrated in Table 1, the N/Si gas ratios of A α-SiCN:H film and N α-SiCN:H film are 1.84 and 2.06, respectively [6]. It implies that more N atoms react with methyl-silane during N α-SiCN:H film deposition. Besides this difference, the A α-SiCN:H film deposited without low frequency RF (LFRF) but N α-SiCN:H film deposited with 920 W LFRF. LFRF would increase ion bombardment during deposition and increase the film density. These two differences may act as factors for increasing the dielectric constant in the N α-SiCN:H film deposition process. 

**Table 2:** Dielectric constant k measurements.

Sample Condition	Thickness		K
	Optical	Electrical	
^0^SiOC:H	387 nm	498 nm	3.04
^1^ A α-SiCN:H	55 nm	44 nm	4.81
^2^ N α-SiCN:H	57 nm	39 nm	5.68
^3^ N α-SiCO:H	30 nm	26 nm	4.46
^4^ PEOX	30 nm	28 nm	4.23
N α-SiCN:H + PEOX + SiOC:H	470 nm	540 nm	3.41
N α-SiCN:H + N α-SiCO:H + SiOC:H	470 nm	544 nm	3.39

^0^: SiOC:H represents organic silicon glass deposition by Applied Material; ^1^: A α-SiCN:H represents nitrogen-doped silicon carbide deposited by Applied Material; ^2^: N α-SiCN:H represents nitrogen-doped silicon carbide deposited by Novellus; ^3^: N α-SiCO:H represents oxygen-doped silicon carbide deposited by Novellus; ^4^: PEOX (plasma enhanced oxide) deposited by Novellus.

**Figure 2:**
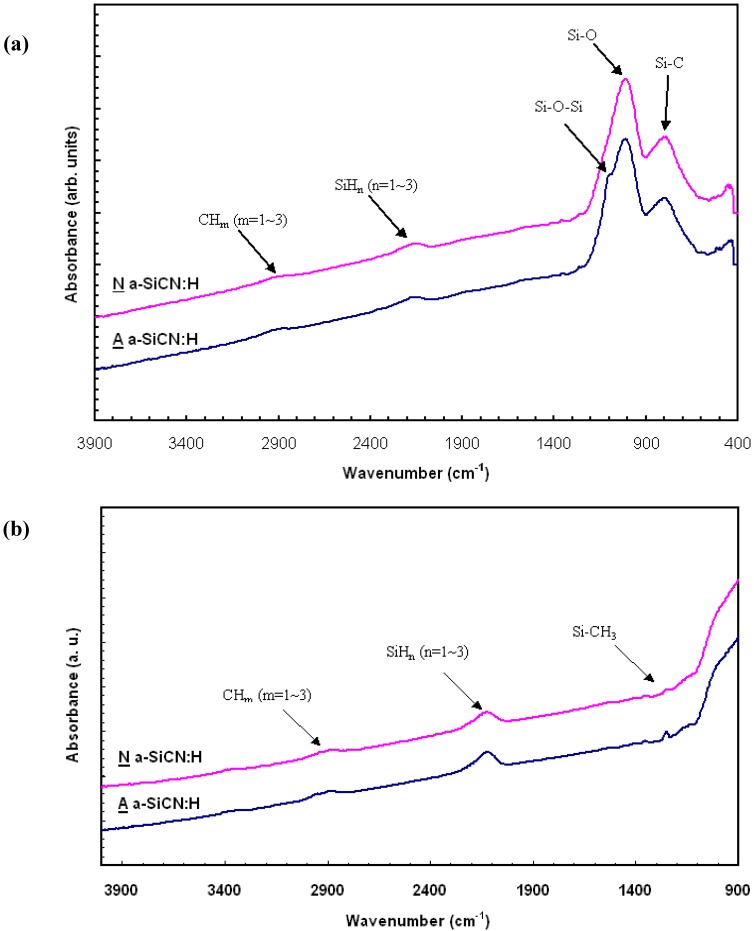
The FTIR spectra of α-SiCN:H film deposited by trimethylsilane (3MS) (**a**) and tetramethylsilane (4MS) (**b**).

**Figure 3:**
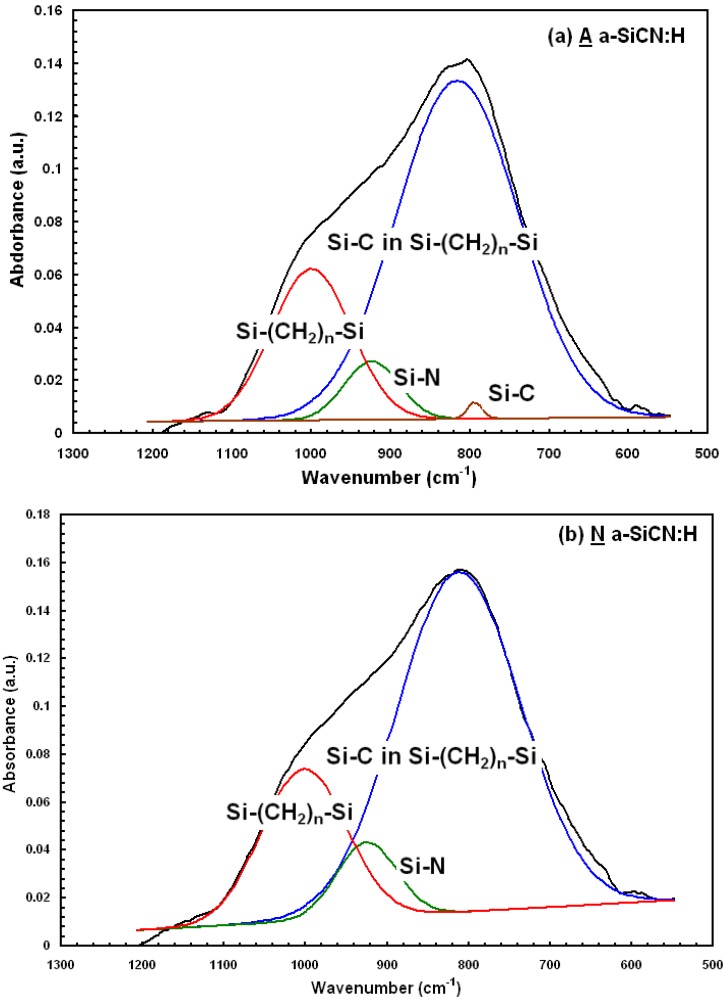
FTIR spectra of α-SiCN:H (**a**) A α-SiCN:H and (**b**) N α-SiCN:H films, which can be split into three peaks, corresponding to Si-C, Si-N, and Si-(CH_2_)_n_-Si, respectively.

Figure 2 shows the FTIR spectroscopy for 3MS A α-SICN:H film and 4MS N α-SICN:H film. The FTIR spectrum serves as an indication of the bond vibration in thin films. For α-SiCN:H film prepared by CVD, the existence of H element makes their FTIR spectrum very complex. The IR spectrum shows absorption peaks at around 1,257, 2,133, 2,900 cm^−1^, corresponding to Si-CH3 stretching, SiHn (n = 1~3), and CHm (m = 1~3) stretching mode, respectively. These spectra suggest that a Si-C network is indeed formed with the incorporation of a large amount of H. From the IR spectra, the peak relative intensity of the Si-CH3 peak for A α-SICN:H film is slightly stronger than the peak for N α-SICN:H film. It implies that the qualitative termination bonding content in N α-SICN:H film is less than in A α-SICN:H film, and gives the N α-SICN:H film a higher density. The reduction in dielectric constant of α-SiCN:H by using 3MS gas is largely due to the decreased density caused by incorporation of Si-CH3 groups. The broader peak of 790–1,020 cm^−1^ shown in Figure 2 can be split into mixed Si-C stretching (796 cm^−1^), Si-C in Si-(CH2)n-Si (814 cm^−1^), Si-N stretching (940 cm^−1^), and Si-(CH2)n-Si (1,000 cm^−1^) vibration mode as shown in Figure 3. Apparently, the A α-SICN:H film showed a small peak of Si-C stretching mode at the wavenumber of 790 cm^−1^ but it was not found in N α-SICN:H film. Besides this variation in bonding configuration, it is believed that the α-SiCN:H film deposited with different precursors would make the film microstructure and properties different.

**Figure 4:**
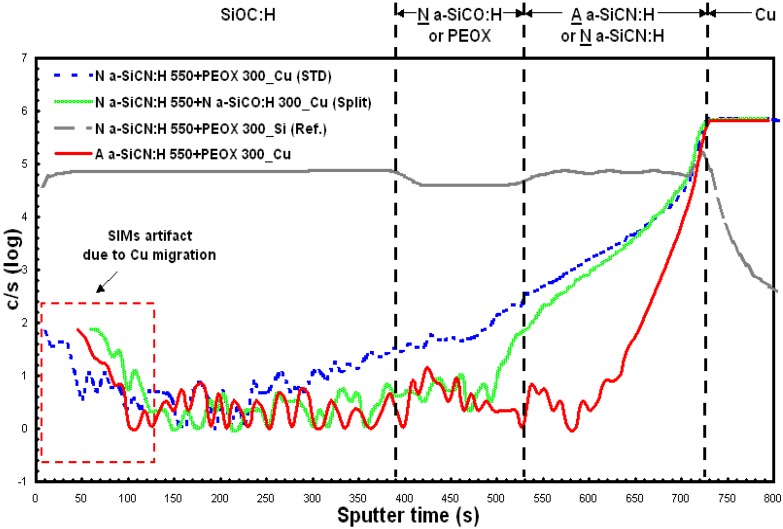
Second Ion Mass Spectroscopy (SIMs) depth profile of Cu in A α-SiCN:H 500+PEOX 300 Cu (red line), N α-SiCN:H 550+PEOX 300 (blue line), and N α-SiCN:H 550+ N α-SiCO:H 300 (green line) dual layer dielectric barrier films.

The copper diffusion barrier properties of the α-SiCN:H films were tested. Analytical characterization was performed using secondary ion mass spectroscopy (SIMs). Figure 4 shows the depth profile of Cu in the testing film stack. Cu is deposited on a blanket wafer using TaN/Ta/Cu seed layer followed by electroplating, and the Cu deposition. For the structure including a dual layer barrier with A α-SICN:H or N α-SICN:H film at the interface between Cu , a 3850A thickness SiOC:H film was deposited.. The dashed line illustrated in Figure 4 is the Si depth profile, which was used to define the boundary of layers for the test sample. A noticeable diffusion of Cu in SiOC:H was observed when α-SiCN:H was deposited using 4MS and PEOX films. However, it could be found that Cu resisted diffusion in A α-SICN:H (red line) film, successfully. This phenomenon was incompatible with the result as mentioned above. On the other hand, there exists large variations in microstructure on α-SiCN:H film deposited by using 3MS or 4MS. In order to resolve the Cu diffusion into SiOC:H film, a new scheme of dual layer barrier was developed. Following the N α-SiCN:H film deposition, a N α-SiCO:H with 300 Å thick film was deposited by using 4MS substitute for PEOX 300A, plasma enhanced oxide. This new scheme of dual layer barrier can resist Cu diffusion into SiOC:H film successfully as shown in Figure 4 (green line).

## 4. Conclusions

The dielectric constant of a measured test film stacking layer composite dual layer diffusion barrier for 65 nm and 45 nm technology has been studied. The dielectric constant of α-SiCN:H film deposited by using 4MS (5.68) is higher than by using 3MS (4.81). From the IR spectra, the termination bond of Si-CH3 has stronger intensity in the A α-SICN:H film, and suggests this would make the film more porous. However, the variation in microstructure for α-SiCN:H films deposited by using 3MS or 4MS can not be identified clearly. On the other hand, the Cu depth profile examined by SIMs showed that Cu would penetrate through the N α-SICN:H film deposited by using 4MS. Conversely, A α-SICN:H film deposited by using 3MS can resist the Cu diffusion into the SiOC:H film successfully. The result is incompatible with the results of dielectric constant and IR spectra. In order to prevent Cu penetration through the barrier film into SiOC:H, a new scheme of N α-SiCN:H/N α-SiCO:H barrier film was developed to substitute the original N α-SICN:H/PEOX barrier film. From the results examined by SIMs, a good performance on Cu diffusion resistance was shown that was consistent with EM and SM testing. In addition, the new scheme can be deposited *in-situ* with the same tool.

## References

[1] Schurr M., Brandl D., Tomaschko Ch., Schoppmann Ch., Voit H. (1995). Langmuir—Blodgett films made from yttrium arachidate. Thin Solid Films.

[2] Chiang C.C., Chen M.C., Ko C.C., Wu Z.C., Jang S.M., Liang M.S. (2003). Physical and barrier properties of plasma-enhanced chemical vapor deposited α-SiC:H films from trimethylsilane and tetramethylsilane. Jpn. J. Appl. Phy..

[3] Chang S.Y., Chang J.Y., Lin S.J., Tsai H.C., Chang Y.S. (2008). Interface chemistry and adhesion strength between porous sioch low-*k* film and sicn layers. J. Electrochem. Soc..

[4] Ishii A., Matsumoto S., Hattori T., Suzuki S., Isono S., Iwasaki A., Tomita K., Hashimoto K., Tawa S., Furusawa T. Interface engineering for highly-reliable 65 nm-node Cu/ULK (k = 2.6) interconnect integration. Proceedings of the IEEE 2005 International Conference.

[5] Chen C.W., Chang T.C., Liu P.T., Tsai T.M., Tseng T.Y. (2005). Effects of oxygen plasma ashing on barrier dielectric SiCN film. Electrochem. Solid-State Lett..

[6] Hatano M., Usui T., Shimooka Y., Kaneko H. EM lifetime improvement of Cu damascene interconnects by p-SiC cap layer. Proceedings of the IEEE 2002 International Conference.

[7] Cui H., Burke P.A. (2004). Time-dependent dielectric breakdown studies of PECVD H:SiCN and H:SiC thin films for copper metallization. J. Electrochem. Soc..

[8] Tsui B.Y., Fang K.L., Lee S.D. (2001). Electrical instability of low-dielectric constant diffusion barrier film (a-SiC:H) for copper interconnect. IEEE Trans. Electron. Devices.

[9] Nakamura N., Takigawa Y., Soda E., Hosoi N., Tarumi Y., Aoyama H., Tanaka Y., Kawamura D., Ogawa S., Oda N., Kondo S., Mori I., Saito S. Design impact study of wiring size and barrier metal on device performance toward 22 nm-node featuring EUV lithography. Proceedings of the Interconnect Technology Conference.

[10] Zhang D.H., Yang L.Y., Li C.Y., Lu P.W., Foo P.D. (2006). Ta/SiCN bilayer barrier for Cu-ultra low *k* integration. Thin Solid Films.

[11] Biggerstaff T.L., Reynolds C.L., Zheleva T., Leis A., Habersat D., Haney S., Ryu S.H., Agarwi A., Duscher G. (2009). Relationship between 4H-SiC/SiO2 transition layer thickness and mobility. Appl. Phys. Lett..

[12] Hu C.K., Rosenberg R., Rathore H.S., Nguyen D.B., Agarwala B. Scaling effect on electromigration in on-chip Cu wiring. Proceedings of the Interconnect Technology.

[13] Chen C.W., Liu P.T., Chang T.C., Yang J.H., Tsai T.M., Wu H.H., Tseng T.Y. (2004). Cu-penetration induced breakdown mechanism for a-SiCN. Thin Solid Films.

